# Development of genome-wide InDel markers and their integration with SSR, DArT and SNP markers in single barley map

**DOI:** 10.1186/s12864-015-2027-x

**Published:** 2015-10-16

**Authors:** Gaofeng Zhou, Qisen Zhang, Cong Tan, Xiao-qi Zhang, Chengdao Li

**Affiliations:** Department of Agriculture and Food, Locked Bag 4, Bentley Delivery Centre, Bentley, WA 6983 Australia; Australian Export Grains Innovation Centre, 3 Baron-Hay Court, South Perth, WA 6155 Australia; Western Barley Genetics Alliance/Centre for Comparative Genomics, Murdoch University, Murdoch, WA 6150 Australia; Western Barley Genetics Alliance/Western Australian State Agricultural Biotechnology Centre, Murdoch University, Murdoch, WA 6150 Australia

**Keywords:** InDel, Barley, High-resolution melting, HRM, SNP, DArT, Genetic map

## Abstract

**Background:**

Development of molecular markers such as SSR (simple sequence repeat), DArT (diversity arrays technology) and SNP (single nucleotide polymorphism) is fundamental for linkage map construction and QTL mapping. However, DArT and SNP genotyping require special tools, and detection of SSR polymorphisms requires time-consuming polyacrylamide electrophoresis. Furthermore, many markers have been mapped in different populations such that their genetic positions are inconsistent. Recently, InDel (insertion and deletion) markers have become popular in genetic map construction and map-based cloning.

**Results:**

Aligning genomic DNA sequences in two barley cultivars (Morex and Barke) identified 436,640 InDels. We designed 1140 InDel markers across the barley genome with an average genetic distance of 1 cM, each having a unique location in the barley genome. High-resolution melting (HRM) technology was used to genotype 55 InDel markers; those PCR amplicons with melting temperature differences >0.3 °C were ideal for HRM genotyping. The 1140 InDel markers together with 383 SSRs, 3909 gene-based SNPs and 1544 DArT markers were integrated into single barley genetic map according to their physical map positions.

**Conclusions:**

High-density InDel markers with specific genome locations were developed, with 6976 molecular markers (SSRs, DArTs, SNPs and InDels) integrated into single barley genetic map. HRM genotyping of the InDel markers each with single PCR band will facilitate quick map construction and gene fine-mapping.

**Electronic supplementary material:**

The online version of this article (doi:10.1186/s12864-015-2027-x) contains supplementary material, which is available to authorized users.

## Background

Traditional markers have played a pivotal role in genetic map construction and marker-assisted selection (MAS) in breeding programs. Genetic maps consist of several types of molecular markers including RFLP (restriction fragment length polymorphism), AFLP (amplified fragment length polymorphism), SSR (simple sequence repeat), STS (sequence-tagged site), DArT (diversity arrays technology) and SNP (single nucleotide polymorphism). RFLP markers have been used to construct first generation genetic maps [1, 2], but such hybridization-based markers have practical disadvantages. This led to interest in PCR-based markers, in particular those based on SSRs. SSR markers were derived from sequences held in public databases including genome sequences and EST sequences [3–5], and small insert genomic libraries [4, 6, 7]. A high-density consensus genetic map containing 775 SSR loci has been constructed in barley [8]. DArT can detect and genotype DNA variations at several hundred genomic loci in parallel without relying on sequence information. DArT markers have been successfully applied to genetic maps and diversity analyses of barley germplasm. The first genetic map consisting of 385 DArT markers was constructed in a population derived from a cross between Steptoe and Morex [9]. These genetic maps were used to construct consensus maps which included RFLPs, SSRs, STSs and DArTs [10, 11]. The first sets of SNP markers were developed by resequencing the European barley gene pool in elite barley genotypes and exploring EST sequences. Based on this information, a pilot oligo nucleotide pool assay containing SNPs in 1524 barley unigenes was developed for use with Illumina Golden Gate Bead Array technology [12, 13]. Later, 3072 SNPs markers were developed based on barley ESTs and sequenced amplicons [14]. A total of 2383 markers including SNP, DArT, SSR and STS markers were mapped in single population [15]. Recently, the genotyping by sequencing (GBS) approach has provided low-cost, high-density genotype information. High-density markers have been mapped in maize, wheat and barley using this technology [16–19]. GBS is a powerful method for developing high-density markers in species without a sequenced genome while providing a genome shotgun sequence.

However, there are disadvantages of these types of markers. DArT genotyping requires special equipment which is unavailable in most research institutions. For SNP markers, although recent advances in molecular techniques have enabled high-throughput SNP genotyping including microarray hybridization, allele-specific PCR detection and primer extension [20–22], and lower-throughput and less equipment dependence including cleaved amplified polymorphic sequence (CAPS) markers [23] and allele-specific PCR primers [24], they are either costly or low-throughput. GBS is high throughput, but costly for barley with its large genome. SSR markers are extensively used in QTL mapping and MAS, however some are nonspecific, or very weak. Furthermore, due to minor differences between genotypes for some SSR markers, laborious sequence-grade high-resolution gels or costly capillary electrophoresis systems are required to genotype these markers.

In contrast to DArT, SSR and SNP markers, InDel markers with moderate polymorphism differences can be amplified using regular PCR instruments and genotyped using an agarose gel electrophoresis system or HRM (high-resolution melting) technology. InDel markers have been successfully used for genetic studies in rice [25] and *Arabidopsis* [26].

HRM curve analysis is a post-PCR analysis method for characterizing nucleic acid samples based on DNA strand dissociation behaviour during transition from double-stranded DNA to single-stranded DNA with increasing temperature. It uses intercalating dyes, highly accurate melt curves and application of specific statistical analyses of genetic variations in PCR amplicons. The amplicon differences are reflected in the melting temperatures. HRM has been used for SNP genotyping and InDel genotyping in wheat [27].

Although InDel markers are advantageous for genetic studies, genome-wide InDel markers have not been explored in barley. The recent release of the barley genomic sequence [28] has made it possible to explore InDel markers in barley. In addition, the published barley physical maps and genetic maps [19, 29] helped us to anchor all available SSRs, DarTs, SNPs and InDel markers to the POPSEQ genetic map and physical map based on sequence similarity.

The objective of this study was to develop genome-wide InDel markers for genetic studies in barley and to use the alternative HRM method to genotype some InDel markers while avoiding post-PCR procedures. Furthermore, these InDel markers were integrated with earlier generation markers, including SSR, DArT and SNP markers, into single map based on barley physical maps instead of the consensus map calculation to avoid genetic position bias.

## Results

### Distribution of InDel markers

All whole genome shotgun (WGS) contig data for Morex, Barke and Bowman was downloaded from ftp://ftpmips.helmholtz-muenchen.de/plants/barley/public_data/. On a genome-wide basis, 436,640 InDels were identified between Morex and Barke in the genomic DNA sequence database. InDels differing by 3–100 bp accounted for 2 % of the total InDels. Our aim was to develop a set of InDel markers with density of one marker per cM, thus about 2000 contigs containing InDels between Morex and Barke distributed evenly in the whole barley genome were selected for primers development. In the 1H and 4H chromosome regions, WGS contigs from the three cultivars (Morex, Barke and Bowman) were aligned. Geneious 6.1.6 was used to develop ~1500 InDel markers with 1–100 bp polymorphisms. Amplicons ranged from 80 to 250 bp. To increase primer specificity, the developed primer sequences were used for BLAST analysis of Morex genomic sequences. A total of 1140 InDel markers were selected as they were anchored specifically to one region of the barley genome (Additional file 1). About 75 % of the total InDel markers had 3–20 bp polymorphisms (Table 1). The number of designed InDel markers in each chromosome ranged from 109 to 241, with densities 1.1 InDels per cM (Table 1).

**Table 1:** The distribution and number of InDel markers in barley

Insertion/Deletion	1H	2H	3H	4H	5H	6H	7H	Total
1–2 bp	12	4	0	6	31	2	0	55
3–10 bp	107	92	84	101	136	57	70	647
11–20 bp	33	26	37	29	36	16	35	212
21–30 bp	12	13	16	14	9	9	8	81
31–40 bp	6	7	6	3	10	7	10	49
41–50 bp	4	4	4	2	2	2	5	23
>50 bp	13	12	6	4	17	13	8	73
Total InDels	187	158	153	159	241	106	136	1140
Length (cM)	142.2	149.2	155.0	115.2	169.4	126.6	140.9	998.4
InDel/cM	1.3	1.1	1.0	1.4	1.4	0.8	1.0	1.1

### Characterization of InDel markers

Of the 1140 InDel markers, 43 were randomly selected from chromosome 5H to test their polymorphisms on 6 % polyacrylamide gel. These markers were developed based on Morex and Barke sequences. These markers, except for InDel5017 and InDel5174, were polymorphic between Morex and Barke (Additional file 2: Table S1), such that ~95 % were successfully developed. We also tested 101 markers from chromosome 1H, 2H, 3H, 6H and 7H, 96 % of these markers have unique amplicon.

### Genotyping by HRM analysis

**Fig. 1:**
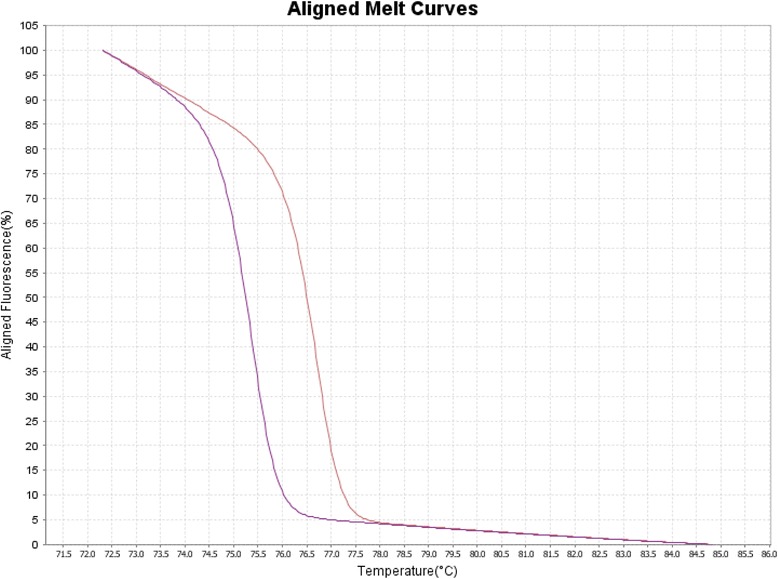
Genotyping InDel5076 using HRM analysis between Morex and Barke. The amplicon size of InDel5076 is 129 and 96 bp in Morex and Barke, respectively. The right melting curve represents Morex and the left melting curve represents Barke

**Fig. 2:**
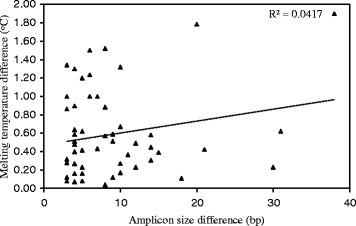
The distribution of melting temperature difference among 55 InDel markers. The melting temperature differences of 55 InDel markers are shown in the figure

Fifty five pairs of InDel primers (Additional file 3: Table S2) each with single PCR product band were used to genotype Morex and Barke using HRM analysis. The results showed that HRM technology can be used to score the two genotypes if the melting temperature difference is >0.3 °C (Figs. 1 and 2). Thirty six pairs of markers were polymorphic (△Tm > 0.3 °C) using HRM technology, such that ~65 % of these markers can be genotyped using this technology (Fig. 2). PCR amplicon differences of these markers between Morex and Barke ranged 3–38 bp (Fig. 2). The coefficient determinant (R^2^) between InDel lengths and melting temperature differences was only 0.0417 (Fig. 2). In other word, there is no correlation between InDel length and melting temperature difference.

Using HRM technology, we tested InDel1064 in a doubled haploid (DH) population containing 360 lines derived from the cross of Alexis and Sloop. The two parental lines had a marker melting temperature difference of 0.6 °C. All of the DH lines were genotyped based on HRM analysis. The PCR product was identified further on 6 % polyacrylamide gel. The gel result was consistent with HRM analysis.

### Integration of SSR, DArT, SNP and InDel markers

**Fig. 3:**
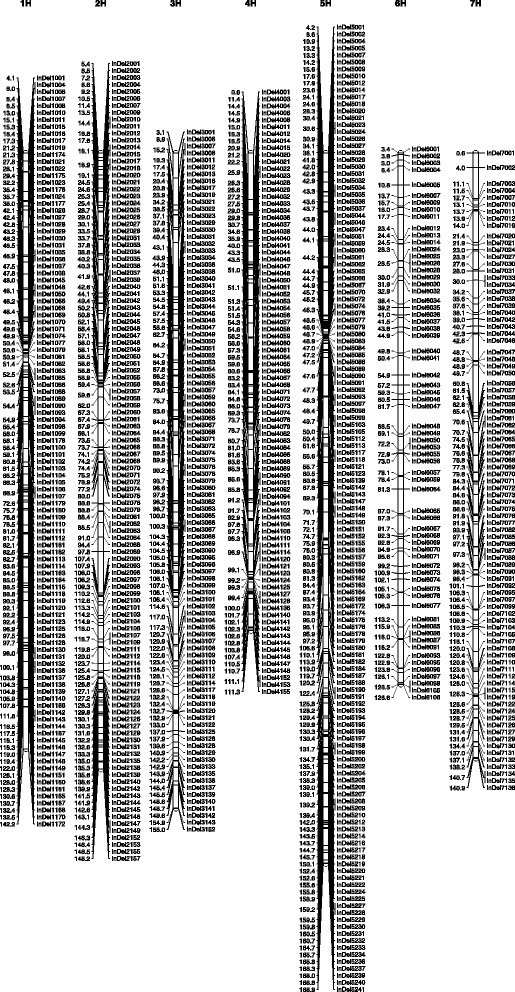
Genetic maps containing InDel markers in barley. Of 1140 InDel markers, 436 were removed due to duplication in the same genetic positions. A total of 704 InDel markers representing 704 loci were used to generate genetic maps. The values on the left of each chromosome represent genetic positions. Markers were placed on the right

The primer sequences of SSR markers and the reference sequence of DArT, SNP and InDel markers were used for BLAST analysis of Morex genomic sequences as described above. The genetic positions of these markers were displayed. The best genetic position was placed on the top. The genetic positions of SSR markers were double checked with the existing genetic map positions with inconsistent SSR markers removed. A total of 6976 markers including 383 SSRs, 1544 DArT, 3909 SNPs and 1140 InDel markers were anchored to the single map (Additional file 1 and Table 2). The distribution of markers on each chromosome was about 1000 markers. The average genetic marker density was about seven markers per cM, i.e. about 700 kb in physical distance between each marker on average. The genetic positions and physical contigs of these markers are listed in Additional file 1, and the genetic map containing InDel markers is presented in Fig. 3.

**Table 2:** Distribution of genetic markers in the barley high-resolution genetic map

Chromosome	SNP	DArT	SSR	InDel	Total
1H	481	182	41	187	891
2H	644	285	66	158	1153
3H	597	256	58	153	1064
4H	450	140	45	159	794
5H	713	214	55	241	1223
6H	463	207	51	106	827
7H	561	260	67	136	1024
Total	3909	1544	383	1140	6976

### Confirmation of InDel marker positions

Thirty-four InDel with three SSR polymorphic markers from chromosomes 1H, 4H and 5H were used to confirm whether InDel markers could be mapped to expected locations in the populations of Alexis × Sloop, Franklin × YYXT and Vlamingh × Buloke, respectively. For the polymorphic InDel markers on chromosome 1H (Alexis × Sloop), all seven InDel markers with one SSR marker were mapped to the 1H region (Additional file 4: Table S3) in the population of Alexis × Sloop. For the fourteen polymorphic InDel markers on chromosome 4H (Franklin × YYXT), all markers were mapped to the 4H region (Additional file 4: Table S3). The linkage map of thirteen InDel markers from chromosome 5H was constructed from the population of Vlamingh × Buloke. All tested 5H markers were mapped to the 5H location (Additional file 4: Table S3). The results indicated that the InDel markers were assigned to the correct chromosomes and marker orders were consistent [19].

## Discussion

In the present study, we developed 1140 InDel markers and confirmed their effectiveness. These InDel markers were integrated with earlier generation SSR, DArT and SNP markers to construct single genetic map containing 6976 markers based on the Morex physical map [19, 28].

### Development of InDel markers

Two types of molecular markers, SSR and DArT, have been widely used in barley genetic map construction and QTL mapping [8, 9, 11, 15]. Recently, sequenced cDNAs have provided a facile route to detect SNPs [14]. However, DArT and SNP markers require special instruments. InDel markers have become a valuable resource for genetics and QTL mapping studies in species such as rice and *Arabidopsis* [25, 26, 30]. Recently, the release of the barley genomic sequence has facilitated the discovery of more polymorphisms within barley cultivars. Abundant and highly reproducible InDel markers were developed based on the alignment of genomic sequences. Unspecific markers were removed after BLAST analysis of the barley genomic DNA sequence. The present study showed that 96 % of InDel markers have unique amplicons. For those markers with multiple bands, the amplicon sizes in Additional file 1 could help to distinguish those amplicons. In rice, a set of 506 InDel markers have size differences larger than 30 bp [25], while most InDel markers in barley ranged from 3 to 20 bp. These InDel markers can be differentiated by HRM or PAGE methods. Mazaheri et al. [31] developed 400,538 PCR-based transposable elements (TE) repeat junctions markers (RJMs) in barley, whereas unlike co-dominant InDel markers, most RJMs were dominant markers.

### Genotyping of InDel markers

InDel markers can be scored using electrophoresis method, such as in *Arabidopsis* [30]. Here we attempted to use an alternative HRM method to score some markers. The differentiation ability of this technique was previously investigated by Mackay et al. [32]. High-resolution amplicon analysis clearly differentiated samples with a 2 bp difference [32]. This study showed that the HRM curve results were consistent with previous studies. Moreover, 55 InDel markers each with single PCR band identified in this study were experimentally confirmed. The InDel genetic map will provide faster and cheaper molecular markers for molecular assisted breeding and map-based cloning in barley.

### Integration of SSR, DArT, SNP and InDel markers

The newly-designed InDel markers were anchored to the Morex physical maps and POPSEQ genetic maps. Combining the physical and genetic maps has been done by Mayer et al. [28] and Mascher et al. [19]. We constructed single map consisting of several types of markers (SSRs, DArT, SNPs and InDels) based on reference sequences of SSR, DArT and SNP markers. Previous consensus map was constructed using model calculations based on existing genetic maps derived from different populations [10], so genetic distance bias occurred with some markers when not shared in all genetic maps. However, in the present study, by BLAST analysis of barley genomic sequences, markers were located to physical positions, and relevant genetic positions were obtained from POPSEQ genetic maps [19, 28].

### Application in QTL mapping and fine mapping

Many QTL traits have been mapped in barley [33, 34]. Developing molecular markers within QTL regions is essential to clone the gene. Morex and Barke are the most commonly used barley genotypes for genetic studies. InDel markers were created based on polymorphisms between these two ecotypes to meet the demands of researchers. In some regions, Bowman contigs were also aligned with Morex and Barke to explore more polymorphic markers.

Some SNP and DArT markers can be converted to InDel markers. By extracting Morex BAC contigs sequences containing SNP and DArT markers (ftp://ftpmips.helmholtz-muenchen.de/plants/barley/public_data/), we can also obtain relevant sequences of Barke and Bowman through the website http://webblast.ipk-gatersleben.de/barley/viroblast.php. InDel markers can be developed in the InDel polymorphic region within the three cultivars (Morex, Barke and Bowman). This strategy will improve the single genetic map. Genomic regions of many SNP and DArT markers can be genotyped without special instruments if converted to InDel markers. In addition, annotated barley genes have been anchored to barley physical maps (http://barleyflc.dna.affrc.go.jp/bexdb/), and the genes within QTL intervals may facilitate QTL fine mapping to determine candidate genes.

## Conclusions

High-density InDel markers with specific genomic locations were developed and 6976 molecular markers (SSRs, DArT, SNPs and InDels) were integrated into single barley genetic map based on barley genomic sequences and POPSEQ genetic maps. Genotyping InDel markers by HRM technology will facilitate quick map construction. The high-density genetic map will be useful for molecular breeding and QTL mapping in barley.

## Methods

### Plant materials and DNA extraction

Ninety-four DH lines derived from a cross of Alexis and Sloop were used to validate some InDel markers on chromosome 1H. A total of 188 F2 lines from a cross between Vlamingh and Buloke were used to validate some polymorphic InDel markers on chromosome 5H, and 172 DH lines from a cross between Franklin and YYXT were used to validate some markers on chromosome 4H. Morex, Barke and Bowman were used to test polymorphism in the InDel markers. Another larger DH population, containing 360 lines derived from the cross of Alexis and Sloop, was used to test InDel1064 with HRM technology. The genomic DNA of each line was isolated from seed using the SDS method described by Ahmed et al. [35] with some modifications. One seed of each line and a steel ball were placed in a 96-well plate, and vibrated with TissueLyser II (Qiagen Co. Ltd) 25 times per second for 5–10 min. We applied 300 μl of extraction buffer (0.1 M Tris–HCl, 0.05 M EDTA, 1.25 % SDS) to each well, and 200 μl supernatant from each well was transferred to a new plate. The plate was kept at 65 °C water bath for 30 min and then centrifuged at 4000 g for 5 min. Then 150 μl supernatant from each well was transferred to a new 96-well plate. Subsequently, 90 μl of isopropanol was applied to each well to precipitate genomic DNA. The genomic DNA pellet was centrifuged to the bottom of each well at 4000 g for 1 min and then washed with 70 % ethanol. Genomic DNA was dried and re-suspended in 100 μl of H_2_O.

### InDel marker development

The genomic DNA sequences of Morex and Barke (verified on 18 Oct 2012) were obtained from ftp://ftpmips.helmholtz-muenchen.de/plants/barley/public_data/. All contigs of Barke were aligned with Morex via BWA with default parameters. In order to ensure the accuracy of sequence alignment, each pair of sequences were realigned with a pairwise aligner software LASTZ [36], then these InDels were extracted with an written Perl script. Finally, filtering was performed under the conditions including the length of both aligning segments bigger than 1 kb, the length of InDels ranging 2–100 bp, the identity between aligning pair of sequences bigger than 95 %, and the distance between each nearby InDels larger than 1 kb. In some loci, the DNA sequence of Bowman was aligned with them. Around 2000 InDel loci, distributed evenly across the barley genome, were selected to design primers. Furthermore, the genetic map of Morex contigs from next-generation sequencing (NGS) (ftp://ftpmips.helmholtz-muenchen.de/plants/barley/public_data/) was obtained from the POPSEQ genetic map [19]. The sequences of Morex and Barke were aligned again in Geneious 6.1.6, and primers developed across the InDel region. Amplicon ranged from 80 to 200 bp. The amplicon can be >200 bp if large InDels exist in the InDel region. To increase the specificity of these primers, primer sequences were used for BLAST analysis of barley genome DNA sequences at http://webblast.ipk-gatersleben.de/barley/viroblast.php. If both forward and reverse primers targeted two or more regions, the markers were removed.

### Characterization of InDel markers with polyacrylamide gel and HRM

We randomly selected 43 InDel markers from chromosome 5H (Additional file 2: Table S1) to test for polymorphisms between Morex and Barke. The 43 markers were synthesized from Sigma-Aldrich. PCRs were conducted in 10 μl reactions containing 5 μl HotStar Taq Master Mix (Qiagen), 0.5 μl of 10 μM primers, 1 μl DNA (100 ng/μl) at 95 °C, 5 min; 34 cycles of (95 °C, 30 s; 56 °C, 30 s; 72 °C, 30 s) and 10 min extension at 72 °C. PCR products were electrophoresed on 6 % polyacrylamide gel. The successful ratio of designed primers was estimated by dividing the total number of polymorphic markers by the total number of markers.

Another 30 selected InDel markers (Additional file 3: Table S2) each with single amplicon were genotyped between Morex and Barke using HRM analysis. PCRs were conducted in 10 μl reactions containing 5 μl SensiFAST™ HRM Kit Buffer (Bioline Pty Ltd), 0.5 μl of 10 μM primers, 1 μl DNA (100 ng/μl) and 3.5 μl H_2_O. PCR plates and films were provided by Life Science (ABI). The reactions were conducted on ViiA7 real-time thermocycle instrument at 95 °C, 5 min; 34 cycles of (95 °C, 30 s; 60 °C, 60 s) and followed by a melting cycle from 60 to 95 °C with 0.025 °C increments every second. The melting temperature was calculated after the PCR reaction with the software ViiA™ 7.

### Integration of SSR, DArT, SNP and InDel markers in single map

Primer sequences of 799 SSR markers [8], genomic sequences of ~2000 DArT markers (http://www.diversityarrays.com/dart-map-sequences), tag sequences of 4608 SNP markers [14] and genomic sequences of InDel markers (ftp://ftpmips.helmholtz-muenchen.de/plants/barley/public_data/) were used for BLAST search in Morex genomic sequence at http://webblast.ipk-gatersleben.de/barley/viroblast.php. The genetic position of DArT, SNP and InDel markers was obtained from BLAST results. The positions with the lowest E-value were placed on top in the results. For SSR markers, both forward and reverse primers were used for BLAST analysis of Morex genomic sequences. The genetic positions of forward and reverse primers were checked manually using existing genetic maps (http://wheat.pw.usda.gov). Only the markers with consistent positions were used to construct single map. Markers that were not localized to barley physical maps or without genetic positions were removed.

### Confirmation of InDel positions

Some InDel markers from chromosomes 1H, 4H and 5H were validated in three populations as described above. Thirty-four InDel and three SSR polymorphic markers (Additional file 4: Table S3) were selected to construct genetic maps. The three SSR markers were used as a reference marker.

### Genetic map construction

For linkage analysis, the genetic distances between markers were analyzed using JoinMap 3.0. The genotype datasets were imported into JoinMap 3.0 to distribute loci into linkage groups. Logarithm of odds (LOD) thresholds (from LOD 3 to LOD 20) were tested to group markers.

Genetic maps were generated with the software MapChart 2.2. Marker names, and genetic map groups and distances were placed in the data area, and genetic maps displayed in the chart area.

### Availability of supporting data

The data sets supporting the results of this article are included within the article and its additional files.

## Additional files

Additional file 1:
**Single genetic map consisting of SSR, DarTs, SNP and InDel markers in barley.** The marker names, physical positions, chromosome positions, primer sequences, amplicon, InDels and references are listed in the file. # means that the markers were not anchored to POPSEQ genetic maps, but could be anchored to Morex physical maps. (XLSX 378 kb) Additional file 2: Table S1. Forty-three randomly-selected InDel markers from chromosome 5H. These markers were genotyped between Morex and Barke. Morex genotype was scored with ‘A’, and Barke genotype was either scored with ‘B’ if different from Morex, or with ‘A’ if the same as Morex. (DOCX 18 kb) Additional file 3: Table S2. Fifty five InDel markers used for HRM analysis between Morex and Barke. The fifty five InDel markers were polymorphic between Morex and Barke, and each marker had single amplicon. HRM analysis of these markers was conducted between Morex and Barke. (DOCX 15 kb) Additional file 4: Table S3. Confirmation of thirty-four InDel and three SSR polymorphic markers positions. The constructed genetic maps were compared with POPSEQ genetic maps. The constructed map positions were listed in the second and third columns. The last two columns show the positions from POPSEQ genetic maps. (DOCX 16 kb)

## Abbreviations

AFLP: Amplified fragment length polymorphism
CAPS: Cleaved amplified polymorphic sequence
DArT: Diversity arrays technology
GBS: Genotyping by sequencing
GWS: Whole genome shotgun
HRM: High-resolution melting
InDel: Insertion and deletion
MAS: Marker-assisted selection
PAGE: Polyacrylamide gel electrophoresis
PCR: Polymerase chain reaction
POPSEQ: Population sequencing
QTL: Quantitative trait loci
RFLP: Restriction fragment length polymorphism
SNP: Single nucleotide polymorphism
SSR: Simple sequence repeat
