# Supplementary material for: Development of genome-wide InDel markers and their integration with SSR, DArT and SNP markers in single barley map
Source: BMC Genomics. 2015 Oct 16;16:804. doi: 10.1186/s12864-015-2027-x (PMC4609152; doi:10.1186/s12864-015-2027-x)
Supplement: Additional file 2: Table S1. — Forty-three randomly-selected InDel markers from chromosome 5H. These markers were genotyped between Morex and Barke. Morex genotype was scored with ‘A’, and Barke genotype was either scored with ‘B’ if different from Morex, or with ‘A’ if the same as Morex. (DOCX 18 kb) [file 12864_2015_2027_MOESM2_ESM.docx]

Table S1 Forty-three randomly-selected InDel markers from chromosome 5H

| Markers | Morex | Barke | Chr. | Pos. (cM) |
| --- | --- | --- | --- | --- |
| InDel5015 | A | B | 5H | 23.61 |
| InDel5017 | A | A | 5H | 24.09 |
| InDel5019 | A | B | 5H | 24.58 |
| InDel5020 | A | B | 5H | 28.26 |
| InDel5021 | A | B | 5H | 30.38 |
| InDel5024 | A | B | 5H | 30.56 |
| InDel5025 | A | B | 5H | 30.56 |
| InDel5026 | A | B | 5H | 30.86 |
| InDel5027 | A | B | 5H | 34.09 |
| InDel5028 | A | B | 5H | 36.11 |
| InDel5029 | A | B | 5H | 41.80 |
| InDel5030 | A | B | 5H | 42.01 |
| InDel5034 | A | B | 5H | 43.26 |
| InDel5046 | A | B | 5H | 43.78 |
| InDel5076 | A | B | 5H | 46.32 |
| InDel5099 | A | B | 5H | 49.65 |
| InDel5100 | A | B | 5H | 49.65 |
| InDel5102 | A | B | 5H | 49.65 |
| InDel5112 | A | B | 5H | 50.44 |
| InDel5143 | A | B | 5H | 69.30 |
| InDel5145 | A | B | 5H | 69.30 |
| InDel5149 | A | B | 5H | 70.14 |
| InDel5150 | A | B | 5H | 71.67 |
| InDel5151 | A | B | 5H | 72.11 |
| InDel5152 | A | B | 5H | 74.65 |
| InDel5156 | A | B | 5H | 76.00 |
| InDel5159 | A | B | 5H | 80.48 |
| InDel5162 | A | B | 5H | 81.32 |
| InDel5163 | A | B | 5H | 84.37 |
| InDel5164 | A | B | 5H | 87.36 |
| InDel5166 | A | B | 5H | 87.36 |
| InDel5168 | A | B | 5H | 87.36 |
| InDel5169 | A | B | 5H | 93.40 |
| InDel5170 | A | B | 5H | 93.40 |
| InDel5171 | A | B | 5H | 93.40 |
| InDel5172 | A | B | 5H | 93.65 |
| InDel5174 | A | A | 5H | 93.89 |
| InDel5175 | A | B | 5H | 95.00 |
| InDel5177 | A | B | 5H | 95.14 |
| InDel5178 | A | B | 5H | 95.90 |
| InDel5179 | A | B | 5H | 97.15 |
| InDel5180 | A | B | 5H | 106.60 |
| InDel5220 | A | B | 5H | 152.36 |
