# Supplementary material for: Development of genome-wide InDel markers and their integration with SSR, DArT and SNP markers in single barley map
Source: BMC Genomics. 2015 Oct 16;16:804. doi: 10.1186/s12864-015-2027-x (PMC4609152; doi:10.1186/s12864-015-2027-x)
Supplement: Additional file 3: Table S2. — Fifty five InDel markers used for HRM analysis between Morex and Barke. The fifty five InDel markers were polymorphic between Morex and Barke, and each marker had single amplicon. HRM analysis of these markers was conducted between Morex and Barke. (DOCX 15 kb) [file 12864_2015_2027_MOESM3_ESM.docx]

Table S2 Fifty five InDel markers used for HRM analysis between Morex and Barke

| Markers | Chr. | Pos.(cM) | InDel (bp) | Melting temperature difference (^o^C) |
| --- | --- | --- | --- | --- |
| InDel1038 | 1H | 47.8 | 10 | 0.67 |
| InDel1052 | 1H | 48.1 | 4 | 0.26 |
| InDel1053 | 1H | 48.1 | 6 | 1.00 |
| InDel1077 | 1H | 50.4 | 9 | 0.51 |
| InDel1091 | 1H | 54.4 | 4 | 0.59 |
| InDel1096 | 1H | 54.9 | 12 | 0.49 |
| InDel2005 | 2H | 9.2 | 10 | 0.17 |
| InDel2058 | 2H | 59.6 | 3 | 0.32 |
| InDel2060 | 2H | 67.3 | 3 | 0.08 |
| InDel2086 | 2H | 91.0 | 3 | 1.00 |
| InDel2095 | 2H | 107.9 | 7 | 1.00 |
| InDel2126 | 2H | 130.1 | 4 | 1.30 |
| InDel2130 | 2H | 132.2 | 5 | 0.23 |
| InDel2135 | 2H | 135.0 | 5 | 1.20 |
| InDel2158 | 2H | 148.2 | 9 | 0.12 |
| InDel3008 | 3H | 15.2 | 15 | 0.39 |
| InDel3013 | 3H | 17.5 | 31 | 0.62 |
| InDel3040 | 3H | 51.1 | 5 | 0.62 |
| InDel3044 | 3H | 54.8 | 4 | 0.27 |
| InDel3063 | 3H | 75.7 | 12 | 0.23 |
| InDel3078 | 3H | 90.2 | 5 | 0.16 |
| InDel3089 | 3H | 104.4 | 4 | 0.40 |
| InDel3098 | 3H | 106.1 | 4 | 0.16 |
| InDel3105 | 3H | 117.3 | 5 | 0.16 |
| InDel3117 | 3H | 128.6 | 8 | 0.04 |
| InDel3151 | 3H | 154.9 | 3 | 0.28 |
| InDel4082 | 4H | 78.7 | 8 | 0.88 |
| InDel4106 | 4H | 97.7 | 11 | 0.37 |
| InDel4132 | 4H | 99.4 | 4 | 0.51 |
| InDel5010 | 5H | 17.6 | 14 | 0.31 |
| InDel5015 | 5H | 23.6 | 8 | 0.57 |
| InDel5017 | 5H | 24.1 | 18 | 0.11 |
| InDel5018 | 5H | 24.6 | 14 | 0.45 |
| InDel5019 | 5H | 24.6 | 4 | 0.90 |
| InDel5021 | 5H | 30.4 | 21 | 0.42 |
| InDel5029 | 5H | 41.8 | 20 | 1.78 |
| InDel5034 | 5H | 43.3 | 3 | 1.34 |
| InDel5048 | 5H | 44.0 | 4 | 0.64 |
| InDel5050 | 5H | 44.0 | 4 | 0.48 |
| InDel5063 | 5H | 44.2 | 6 | 1.23 |
| InDel5070 | 5H | 45.7 | 5 | 0.41 |
| InDel5078 | 5H | 46.5 | 7 | 0.43 |
| InDel5240 | 5H | 168.8 | 3 | 0.86 |
| InDel6003 | 6H | 5.0 | 5 | 0.08 |
| InDel6019 | 6H | 24.5 | 4 | 0.07 |
| InDel6025 | 6H | 28.5 | 8 | 1.52 |
| InDel6085 | 6H | 115.9 | 10 | 1.32 |
| InDel6104 | 6H | 126.5 | 14 | 0.58 |
| InDel7007 | 7H | 12.7 | 9 | 0.59 |
| InDel7022 | 7H | 21.9 | 4 | 0.16 |
| InDel7030 | 7H | 27.6 | 30 | 0.23 |
| InDel7047 | 7H | 48.7 | 10 | 0.27 |
| InDel7049 | 7H | 48.9 | 38 | 1.90 |
| InDel7099 | 7H | 106.5 | 3 | 0.12 |
| InDel7136 | 7H | 140.9 | 6 | 1.50 |
