# Supplementary material for: Development of genome-wide InDel markers and their integration with SSR, DArT and SNP markers in single barley map
Source: BMC Genomics. 2015 Oct 16;16:804. doi: 10.1186/s12864-015-2027-x (PMC4609152; doi:10.1186/s12864-015-2027-x)
Supplement: Additional file 4: Table S3. — Confirmation of thirty-four InDel and three SSR polymorphic markers positions. The constructed genetic maps were compared with POPSEQ genetic maps. The constructed map positions were listed in the second and third columns. The last two columns show the positions from POPSEQ genetic maps. (DOCX 16 kb) [file 12864_2015_2027_MOESM4_ESM.docx]

Table S3 Confirmation of thirty-four InDel and three SSR polymorphic markers positions

| Markers | Chr. | Pos.(cM) | Chr. (POPSEQ) | POPSEQ (cM) |
| --- | --- | --- | --- | --- |
| InDel1033 | 1H | 48.1 | 1H | 46.5 |
| InDel1042 | 1H | 48.1 | 1H | 47.8 |
| InDel1040 | 1H | 58.1 | 1H | 47.8 |
| InDel1063 | 1H | 48.1 | 1H | 48.1 |
| GBM1234 | 1H | 48.1 | 1H | 48.1 |
| InDel1062 | 1H | 48.1 | 1H | 48.1 |
| InDel1064 | 1H | 48.1 | 1H | 48.1 |
| InDel1080 | 1H | 48.1 | 1H | 50.6 |
| InDel4089 | 4H | 80.0 | 4H | 85.3 |
| InDel4085 | 4H | 80.0 | 4H | 80.9 |
| InDel4084 | 4H | 85.6 | 4H | 80.9 |
| InDel4091 | 4H | 89.0 | 4H | 85.6 |
| InDel4087 | 4H | 92.0 | 4H | 81.6 |
| InDel4112 | 4H | 95.0 | 4H | 98.8 |
| InDel4116 | 4H | 97.0 | 4H | 98.9 |
| InDel4117 | 4H | 97.0 | 4H | 98.9 |
| InDel4121 | 4H | 100.0 | 4H | 99.1 |
| InDel4114 | 4H | 100.0 | 4H | 98.9 |
| InDel4130 | 4H | 105.0 | 4H | 99.4 |
| GBM1220 | 4H | 105.0 | 4H | 99.4 |
| InDel4131 | 4H | 108.0 | 4H | 99.4 |
| InDel4128 | 4H | 108.0 | 4H | 99.4 |
| InDel4132 | 4H | 112.0 | 4H | 99.4 |
| InDel5039 | 5H | 41.2 | 5H | 43.7 |
| Bmag0751 | 5H | 41.2 | 5H | 43.8 |
| InDel5045 | 5H | 41.2 | 5H | 43.8 |
| InDel5055 | 5H | 41.2 | 5H | 44.1 |
| InDel5058 | 5H | 41.2 | 5H | 44.1 |
| InDel5059 | 5H | 41.2 | 5H | 44.1 |
| InDel5065 | 5H | 41.2 | 5H | 44.4 |
| InDel5067 | 5H | 41.2 | 5H | 44.9 |
| InDel5070 | 5H | 41.2 | 5H | 45.7 |
| InDel5073 | 5H | 41.2 | 5H | 46.2 |
| InDel5085 | 5H | 44.4 | 5H | 47.2 |
| InDel5086 | 5H | 44.4 | 5H | 47.5 |
| InDel5128 | 5H | 53.4 | 5H | 60.5 |
| InDel5142 | 5H | 86.9 | 5H | 67.8 |
